# Efficient Circuit Implementations of Continuous-Time Quantum Walks for Quantum Search

**DOI:** 10.3390/e27050454

**Published:** 2025-04-23

**Authors:** Renato Portugal, Jalil Khatibi Moqadam

**Affiliations:** National Laboratory of Scientific Computing (LNCC), Av. Getulio Vargas 333, Petrópolis 25651-075, Brazil

**Keywords:** quantum computer, quantum walk, spatial search algorithm, complete graph, bipartite graph, hypercube

## Abstract

Quantum walks are a powerful framework for simulating complex quantum systems and designing quantum algorithms, particularly for spatial search on graphs, where the goal is to find a marked vertex efficiently. In this work, we present efficient quantum circuits that implement the evolution operator of continuous-time quantum-walk-based search algorithms for three graph families: complete graphs, complete bipartite graphs, and hypercubes. For complete and complete bipartite graphs, our circuits exactly implement the evolution operator. For hypercubes, we propose an approximate implementation that closely matches the exact evolution operator as the number of vertices increases. Our Qiskit simulations demonstrate that even for low-dimensional hypercubes, the algorithm effectively identifies the marked vertex. Furthermore, the approximate implementation developed for hypercubes can be extended to a broad class of graphs, enabling efficient quantum search in scenarios where exact implementations are impractical.

## 1. Introduction

The continuous-time quantum walk (CTQW) was introduced by Farhi and Gutmann [1] as the quantum version of the continuous-time Markov chain, and this new model proved useful to build quantum algorithms, such as algorithms for spatial search on graphs [2,3,4,5,6] and NAND-based formula evaluation [7]. CTQWs are also universal for quantum computation, meaning that any quantum algorithm can be efficiently simulated using a continuous-time quantum walk [8,9,10]. Experimental implementations of search algorithms using continuous-time quantum walks are described in [11,12,13,14,15].

Circuits for the implementation of CTQWs on graphs with no marked vertex are obtained using Hamiltonian simulation—for instance, Refs. [16,17,18] describe circuits for the CTQW on circulant graphs using this method. Hamiltonian simulation is different from the methods used to implement discrete-time quantum walks [19,20], such as the ones described in [20,21,22,23,24].

In this work, we tackle the problem of finding efficient circuits for the implementation of CTQW-based search algorithms on graphs with one marked vertex. We use a Hamiltonian simulation based on efficient state preparation. We focus on three graph classes: complete graphs, complete bipartite graphs, and hypercubes. The techniques described here can be applied to other graph classes. When the graph structure is simple enough, such as complete graphs and complete bipartite graphs, we build a circuit that simulates the exact evolution operator *U*, modulo a global phase. On the other hand, for hypercubes, we build a circuit that implements a unitary operator $U^{\prime }$ that simulates *U* approximately, not in the sense that the Hilbert–Schmidt distance between *U* and $U^{\prime }$ is small, but in the sense that the difference between the probability of finding the marked vertex using *U* and $U^{\prime }$ is small when the initial condition is the uniform superposition |s〉 of all states of the computational basis.

Our method relies on the fact that, for some graph classes, the fidelity between the uniform state |s〉 with the plane spanned by the ground state $|\lambda^{-}\rangle$ and first excited state $|\lambda^{+}\rangle$ of the Hamiltonian tends to one asymptotically (N→∞, where *N* is the number of vertices) [25]. For these graph classes, we describe a method to calculate approximations of $|\lambda^{\pm }\rangle$. Since the Hamiltonian is described only in terms of projectors on the eigenspaces of these two eigenvectors, we use the fact that they commute, and then the evolution operator can be written as a product of two unitary operators $U=U_{\lambda^{+}}U_{\lambda^{-}}$. In the final step, we implement $U_{\lambda^{+}}$ and $U_{\lambda^{-}}$ using circuits for state preparation, that is, using the circuit of an operator $A_{\lambda }$ such that $A_{\lambda }|0\rangle =|\lambda \rangle$, where |0〉 is the first state of the computational basis.

In all graph classes that we have addressed, we have obtained circuits for the evolution operators with $O(\log^{2}N)$ basic gates. However, to run the whole search algorithm we need $O(\sqrt{N})$ steps—that is, at the end, we implement $U^{\left\lfloor t_{\mathrm{opt}}\right\rfloor }$, where $t_{\mathrm{opt}}$ is the optimal running time of the algorithm. Therefore, the circuits have $\tilde{O}\left(\sqrt{N}\right)$ basic gates.

Compared to previous works on Hamiltonian simulation of quantum walks [12,13,14], our contribution lies in providing explicit and efficient quantum circuits tailored specifically for CTQW-based search algorithms, rather than just for CTQWs without marked vertices. Furthermore, we introduce a method that allows for the construction of simpler circuits for hypercubes and complete bipartite graphs, which can be extended to a broad class of graphs.

The structure of the paper is as follows. Section 2 reviews the continuous-time quantum walk dynamics on graphs. The following sections present the decomposition of the evolution operator and the associated circuit for two kinds of CTQWs on graphs, namely, graphs with no marked vertex, and graphs with one marked vertex. We address three graph classes: (1) complete graphs in Section 3, (2) complete bipartite graphs in Section 4, and (3) hypercubes in Section 5. In Section 6, we present our conclusions.

## 2. CTQW on Graphs

A continuous-time quantum walk [1,26] on a graph Γ(V,E), with vertex set *V* and edge set *E*, is a quantum dynamics in which the state space is associated with *V*, and the evolution operator is $U\left(t\right)=e^{-iHt}$, where H=−γA is the Hamiltonian, γ is a positive constant setting the transition rate of the walker, and *A* the adjacency matrix, which is a symmetric matrix whose entries $A_{k\ell }$ are 1 for all pairs of vertices $(v_{k},v_{\ell })\in E$, and 0 otherwise. If the initial state is |ψ(0)〉, the state of the quantum walk at time *t* is |ψ(t)〉=U(t)|ψ(0)〉.

### 2.1. Spatial Search on Graphs

The CTQW is an interesting framework for developing spatial search algorithms on graphs. These algorithms aim to find a marked vertex w∈V as quickly as possible starting from an initial state uniformly spread over all vertices. The standard recipe is driven by the modified following Hamiltonian [2]:

(1) H=−γA−|w〉〈w|.

The CTQW-based search provides a quadratic speedup when compared with a random-walk-based search on a complete graph [2], bipartite graph, hypercube [2], and Johnson graph, to mention a few of them. However, the CTQW-based search algorithms do not outperform the classical algorithms on *d*-dimensional lattices with d<4 [2].

The efficiency of the algorithm is determined by the optimal running time $t_{\mathrm{opt}}$ so that the success probability $p_{\mathrm{succ}}={\left|\langle w|\psi \left(t_{\mathrm{opt}}\right)\rangle \right|}^{2}$ is as large as possible, where

(2) $$|\psi \left(t\right)\rangle ={\left(e^{-iH}\right)}^{t}|\psi \left(0\right)\rangle ,$$

in which H is the modified Hamiltonian (1), and |ψ(0)〉 is the initial condition

(3) $$|\psi \left(0\right)\rangle =|s\rangle =\frac{1}{\sqrt{N}}\sum_{j=0}^{N-1}|j\rangle ,$$

where *N* is the number of vertices.

### 2.2. Locality

Quantum walk is a subarea of quantum mechanics that is characterized essentially by two aspects: (1) the allowed locations of the walker is a spatial discrete structure, usually modeled by a graph, and (2) the evolution operator is local in the sense that if the walker is on vertex *v*, the walker hops to the neighboring vertices of *v* before reaching the other vertices. The dynamics given in  (2) satisfies the two aspects via the following reasons: Let the initial condition be |ψ(0)〉=|v〉 and take γ constant for all graphs in a graph class. After an infinitesimal time ϵ, the locations of the walker is the superposition state

(4) $$|v\rangle +\epsilon \left(\gamma A+\delta_{v,w}\right)|v\rangle +O\left(\epsilon^{2}\right)|v\rangle ,$$

which satisfies the locality aspect because the action of the adjacency matrix on |v〉 results in a superposition of vertices in the neighborhood of *v*.

If we want to compare the time complexity of CTQW-based with random-walk-based search algorithms, we have to change the viewpoint. Since the random walk evolves in discrete time-steps, a fair comparison demands that we take t=1 in (2) and repeat the action of $e^{-iH}$ over and over. To satisfy the locality aspect in this case, we take γ small, typically $O\left(1/N\right)$, where *N* is the number of vertices. The optimal value of γ of the CTQW-based search algorithm on the class of complete graphs and complete bipartite graphs is $O\left(1/N\right)$. On the other hand, the optimal value of γ for the class of hypercube graphs is $O\left(1/\log N\right)$, which satisfies a weaker version of the locality aspect.

## 3. Complete Graph

Let $K_{N}$ be the complete graph, where $N=2^{n}$. The Hilbert space associated with $K_{N}$ is spanned by the vertices. The adjacency matrix of $K_{N}$ is

(5) A=N|s〉〈s|−I,

where |s〉 is the uniform superposition of all vertices or all basis states, $|s\rangle =H^{⊗n}|0\rangle ,$ and $H=\frac{1}{\sqrt{2}}\left(\begin{array}{cc} 1 & \;1\\ 1 & -1 \end{array}\right)$ is the Hadamard operator. Assuming that γ=1/N, the evolution operator of a CTQW on $K_{N}$ with no marked vertex is

(6) $$U\left(t\right)=e^{\frac{-it}{N}}H^{⊗n}e^{it|0\rangle \langle 0|}H^{⊗n}.$$

Using the identity

(7) $$e^{itP}=I+\left(e^{it}-1\right)P,$$

which is true for any orthogonal projector *P*, we obtain

(8) $$U\left(t\right)=e^{\frac{-it}{N}}H^{⊗n}\left(I+\left(e^{it}-1\right)\left|0\rangle \langle 0\right|\right)H^{⊗n}.$$

**Figure 1:**
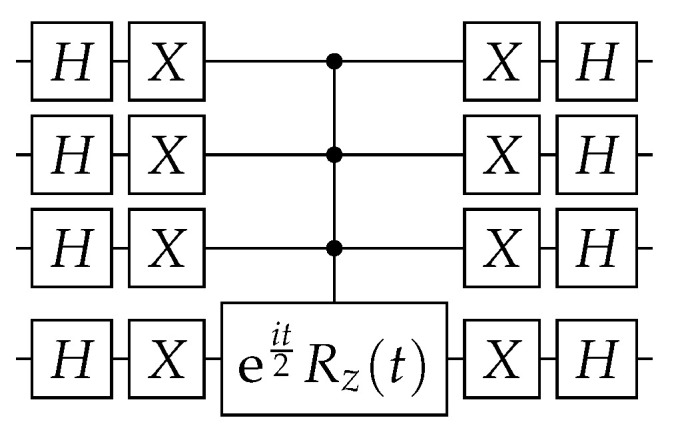
Circuit implementing the CTQW on the complete graph $K_{N}$, where U(t) is given by Equation (8) and N=16.

The circuit that implements U(t) up to a global phase is depicted in Figure 1 for the case n=4. The implementation of

(9) $$R=I+\left(e^{it}-1\right)\left|0\rangle \langle 0\right|$$

uses a multi-controlled gate

(10) $$R_{z}\left(\theta \right)=\left(\begin{array}{cc} e^{-i\theta /2} & \;0\\ 0 & e^{i\theta /2} \end{array}\right)$$

multiplied by a relative phase $e^{\frac{i\theta }{2}}$. Note that $R|0\rangle =e^{it}|0\rangle$ and R|j〉=|j〉 if j≠0. If n=1, *R* is obtained as follows:

(11) $$R=e^{\frac{it}{2}}XR_{z}\left(t\right)X=\left(\begin{array}{cc} e^{it} & 0\\ 0 & 1 \end{array}\right).$$

If n>1, we have to use n−1 control qubits that are activated only when they are set to 0.

In Qiskit (https://qiskit.org/ (accessed on 19 March 2025)), the multi-controlled gate $e^{\frac{it}{2}}R_{z}\left(t\right)$ is implemented using gate mcrz (see package *qiskit.circuit.QuantumCircuit*), and its decomposition in terms of universal gates is automatically provided.

### Spatial Search on Complete Graph

We take γ=1/N because this is the asymptotically optimal value for the spatial search algorithm on the complete graph $K_{N}$ [2]. This choice is made for the evolution under $e^{-iH}$, where the hopping rate is γ=1/N. Note that, assuming *N* is large, this choice ensures the locality of the dynamics in the sense discussed in Section 2.2. Using this value in the modified Hamiltonian (1), we obtain

(12) $$H=\frac{I}{N}-\left|s\rangle \langle s\right|-\left|w\rangle \langle w\right|$$

where *w* is the marked vertex. Alternatively, we write

(13) $$H-\frac{I}{N}=\lambda^{+}|\lambda^{+}\rangle \langle \lambda^{+}|+\lambda^{-}|\lambda^{-}\rangle \langle \lambda^{-}|,$$

where $|\lambda^{\pm }\rangle$ are the only eigenvectors of H−I/N associated with non-zero eigenvalues $\lambda^{\pm }$, which are given by

(14) $$\lambda^{\pm }=-1\pm \frac{1}{\sqrt{N}}$$

and the entries of the eigenvectors are

(15) $$\langle j|\lambda^{\pm }\rangle =\left\{\begin{array}{cc} \frac{\sqrt{-\lambda^{\pm }}}{\sqrt{2}} & \mathrm{if}\;j=w,\\ \frac{\mp 1}{\sqrt{-2N\lambda^{\pm }}} & \mathrm{if}\;j\neq w. \end{array}\right.$$

In the continuation, we assume that w=0. The eigenvectors in this case are

(16) $$|\lambda^{\pm }\rangle =\frac{1}{\sqrt{-2N\lambda^{\pm }}}\left(\begin{array}{c} -\sqrt{N}\lambda^{\pm }\\ \mp 1\\ \vdots \\ \mp 1 \end{array}\right).$$

Using that the projectors $|\lambda^{+}\rangle \langle \lambda^{+}|$ and $|\lambda^{-}\rangle \langle \lambda^{-}|$ commute, the evolution operator of a CTQW on $K_{N}$ with one marked vertex is

(17) $$U\left(t\right)=e^{-it/N}e^{-it\lambda^{+}|\lambda^{+}\rangle \langle \lambda^{+}|}e^{-it\lambda^{-}|\lambda^{-}\rangle \langle \lambda^{-}|}.$$

Using Equation (7), we obtain

(18) $$U\left(t\right)=e^{-it/N}A_{\lambda^{+}}R_{\lambda^{+}}\left(t\right)A_{\lambda^{+}}^{†}A_{\lambda^{-}}R_{\lambda^{-}}\left(t\right)A_{\lambda^{-}}^{†}$$

where

(19) $$R_{\lambda^{\pm }}\left(t\right)=I+\left(e^{-it\lambda^{\pm }}-1\right)\left|0\rangle \langle 0\right|$$

and (the choice of |0〉 in this equation has no relation with the location of the marked vertex)

(20) $$A_{\lambda^{\pm }}|0\rangle =|\lambda^{\pm }\rangle .$$

**Figure 2:**
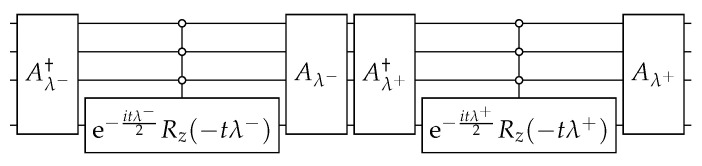
The circuit that implements U(t) (18) on $K_{2^{n}}$ with one marked vertex when n=4. The circuit of $A_{\lambda^{\pm }}$ is depicted in Figure 3.

**Figure 3:**
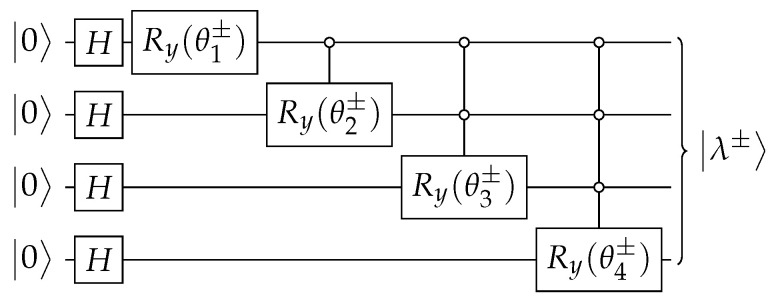
The circuit that implements $A_{\lambda^{\pm }}$ (20) when n=4. Angles $\theta_{k}^{\pm }$ are given by Equation (21).

The circuit that implements U(t) (18) is depicted in Figure 2. The circuit of $A_{\lambda^{\pm }}$ is obtained using the techniques described in Appendix A and is depicted in Figure 3. Using Equations (A3), (A5), and (A9), the angles of operators $R_{y}$ when w=0 are

(21) $$\theta_{k}^{\pm }=\mp 2\arctan\frac{1}{\sqrt{1+2^{k}\mp \frac{2^{k+1}}{\sqrt{N}}}}-\frac{\pi }{2},$$

for 1≤k≤n. When w≠0, we must implement the modifications described at the end of Appendix A.

If we consider a finite set of basic gates, the number of basic gates required to implement the $A_{\lambda^{\pm }}$ in Figure 3 is $O(n^{2})$ for the angles of Equation (21). The computational cost to implement $R_{\lambda^{\pm }}\left(t\right)$ depends on *t*. For a fixed *t*, say t=1, the number of universal gates is also $O(n^{2})$ because $\lambda^{\pm }\approx -1$ for large *N*. For the search algorithm, the optimal running time is the following [2]:

(22) $$t_{\mathrm{opt}}=\frac{\pi }{2}\sqrt{N},$$

which means that the success probability is exactly 1 at $t_{\mathrm{opt}}$. In terms of oracle-based algorithms, the oracle is the circuit of $e^{-iH}$, obtained by taking t=1 in the circuit of Figure 2. To find the marked vertex, in this case w=0, one must concatenate the circuit of Figure 2$\left\lfloor t_{\mathrm{opt}}\right\rfloor$ times. Therefore, the implementation requires $\tilde{O}\left(\sqrt{N}\right)$ basic gates. Note that when *N* is large, the operator $e^{-iH}$ is local in the sense discussed in Section 2.2. The walker hops only to neighboring vertices after the action of $e^{-iH}$ because the hopping rate is γ=1/N. To compare the continuous-time evolution with a discrete-time random-walk-based evolution or with Grover’s algorithm, we have to repeat the action of $e^{-iH}$ over and over instead of setting $t=\pi \sqrt{N}/2$ in the circuit of Figure 2 (see discussion in Section 2.2).

**Figure 4:**
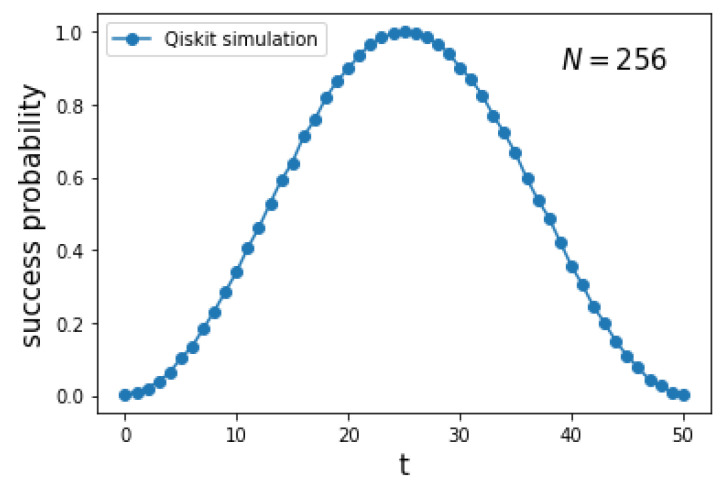
Success probability ${\left|\langle 0|\psi (t)\rangle \right|}^{2}$ as a function of the number of steps *t* for the search on $K_{2^{n}}$ with n=8 using a Qiskit implementation of the circuit of Figure 2.

Figure 4 depicts the success probability ${\left|\langle 0|\psi (t)\rangle \right|}^{2}$ as a function of the number of steps *t* for a complete graph $K_{256}$. The curve is obtained using the implementation of the circuit of Figure 2 in Qiskit. Since *t* is discrete, the optimal running time is $\left\lfloor t_{\mathrm{opt}}\right\rfloor$.

## 4. Complete Bipartite Graph

Let $K_{n,n}$ be a complete bipartite graph with $N=2n=2^{m}$ vertices and adjacency matrix *A*. The Hilbert space associated with $K_{n,n}$ is spanned by the vertices. Define $\tilde{A}$ as

(23) $$\tilde{A}=H^{⊗m}AH^{⊗m},$$

which is a remarkably simple matrix given by

(24) $$\tilde{A}=n\;|0\rangle \langle 0|-n\;|n\rangle \langle n|,$$

whose nonzero eigenvalues are $\lambda^{\pm }=\pm n$ with associated eingenvectors $|\lambda^{+}\rangle =|0\rangle$ and $|\lambda^{-}\rangle =|n\rangle$. Taking γ=1/n and using the fact that the projectors |0〉〈0| and |n〉〈n| commute, the evolution operator of a CTQW on the complete bipartite graph with no marked vertex is

(25) $$\begin{array}{cc} U(t) & =e^{i\gamma At}=H^{⊗m}e^{i\gamma \tilde{A}t}H^{⊗m}\\ \; & =H^{⊗m}e^{it|0\rangle \langle 0|}e^{-it|n\rangle \langle n|}H^{⊗m}. \end{array}$$

Using Equation (7), we obtain

(26) $$U\left(t\right)=H^{⊗m}R_{0}R_{n}H^{⊗m},$$

where

$$\begin{array}{cc} R_{0} & =I+\left(e^{it}-1\right)|0\rangle \langle 0|,\\ R_{n} & =I+\left(e^{-it}-1\right)|n\rangle \langle n|. \end{array}$$

**Figure 5:**
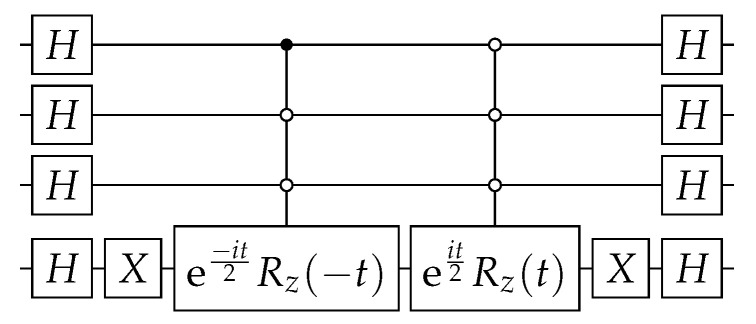
Circuit implementing the CTQW on the bipartite graph $K_{n,n}$ with n=8, where U(t) is given by Equation (26).

The circuit that implements U(t) up to a global phase is depicted in Figure 5 for the case m=4. Note that the first control qubit of $R_{n}$ is solid because $n={\left(10...0\right)}_{2}$.

### Spatial Search on Complete Bipartite Graph

We take γ=1/n because this is the asymptotically optimal value for the spatial search algorithm on the complete bipartite graph $K_{n,n}$, as can be derived using the method described in Appendix B. Note that, assuming *n* is large, this choice of γ ensures the locality of the dynamics in the sense discussed in Section 2.2. Using γ=1/n in the modified Hamiltonian (1), we obtain

(27) $$H=-H^{⊗m}\left(|0\rangle \langle 0|-|n\rangle \langle n|\right)H^{⊗m}-\left|w\rangle \langle w\right|$$

where *w* is the marked vertex. Since this Hamiltonian has only three eigenvalues different from 0, we write

(28) $$H=\lambda^{+}|\lambda^{+}\rangle \langle \lambda^{+}|+\lambda^{-}|\lambda^{-}\rangle \langle \lambda^{-}|+\lambda_{0}|\lambda_{0}\rangle \langle \lambda_{0}|,$$

where $\lambda^{-}<\lambda^{+}<\lambda_{0}$ are solutions of

(29) $$\lambda^{3}+\lambda^{2}-\lambda -\left(1-\frac{1}{n}\right)=0.$$

When w=0, the entries $\lambda \left(j\right)=\langle j|\lambda \rangle$ of associated eigenvectors are

(30) $$\lambda \left(j\right)=\left\{\begin{array}{cc} \frac{1}{\sqrt{c}} & \mathrm{if}\;j=0,\\ \frac{a}{\sqrt{c}} & \mathrm{if}\;1\leq j\leq n,\\ \frac{b}{\sqrt{c}} & \mathrm{if}\;n<j<N, \end{array}\right.$$

where

(31) $$\begin{array}{cc} a & =\frac{n\lambda^{2}+n\lambda -1}{n-1}, \end{array}$$

(32) $$\begin{array}{cc} b & =-1-\lambda , \end{array}$$

(33) $$\begin{array}{cc} c & =1+\left(n-1\right)a^{2}+nb^{2}. \end{array}$$

Using the fact that the projectors $|\lambda^{+}\rangle \langle \lambda^{+}|$, $|\lambda^{-}\rangle \langle \lambda^{-}|$, and $|\lambda_{0}\rangle \langle \lambda_{0}|$ commute, the evolution operator of a CTQW on $K_{n,n}$ with one marked vertex is

(34) $$U\left(t\right)=e^{-it\lambda_{0}|\lambda_{0}\rangle \langle \lambda_{0}|}e^{-it\lambda^{+}|\lambda^{+}\rangle \langle \lambda^{+}|}e^{-it\lambda^{-}|\lambda^{-}\rangle \langle \lambda^{-}|}.$$

Using Equation (7), we obtain

(35) $$U\left(t\right)=A_{\lambda_{0}}R_{\lambda_{0}}A_{\lambda_{0}}^{†}A_{\lambda^{+}}R_{\lambda^{+}}A_{\lambda^{+}}^{†}A_{\lambda^{-}}R_{\lambda^{-}}A_{\lambda^{-}}^{†}$$

where

(36) $$R_{\lambda }=I+\left(e^{-it\lambda }-1\right)\left|0\rangle \langle 0\right|$$

and

(37) $$A_{\lambda }|0\rangle =|\lambda \rangle ,$$

where $\lambda \in \{\lambda^{\pm },\lambda_{0}\}$ is a root of (29) and |λ〉 is the associated eigenvector.

**Figure 6:**
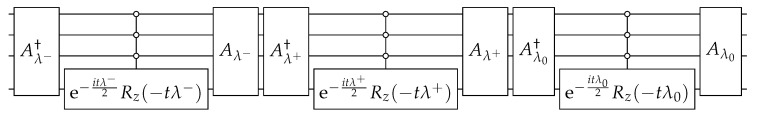
The circuit that implements U(t) (35) on $K_{n,n}$ with one marked vertex when n=8. The circuit of $A_{\lambda }$ is depicted in Figure 7.

**Figure 7:**
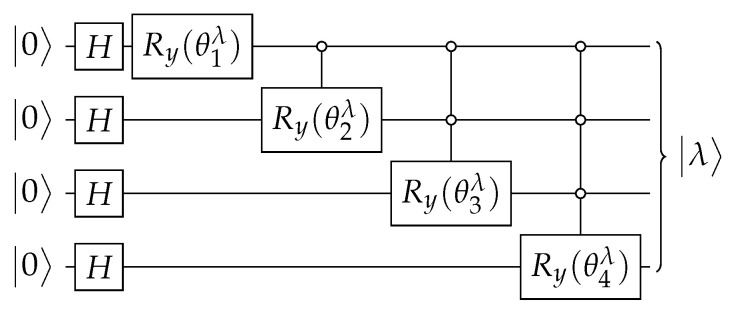
The circuit that implements $A_{\lambda }$ (37) when m=4. Angles $\theta_{k}^{\lambda }$ are given by Equation (38).

The circuit that implements U(t) (35) is depicted in Figure 6. The circuit that implements $A_{\lambda }$ when |λ〉 is given by (30) is obtained using the techniques described in Appendix A and is depicted in Figure 7. Using Equations (A3), (A5), and (A9), the angles of operators $R_{y}$ when w=0 are

(38) $$\begin{array}{cc} \theta_{k}^{\lambda }=\; & 2\arctan\frac{a\left(1-\delta_{km}\right)+b\delta_{km}}{\sqrt{a^{2}\left(1-2^{k-1}\right)-b^{2}2^{k-1}+c2^{k-m}}}-\frac{\pi }{2}, \end{array}$$

for 1≤k≤m. Note that *a*, *b*, and *c* depend on λ, which assumes the three roots of Equation (29). When w≠0, we must implement the modifications described at the end of Appendix A.

If we remove the gates of the circuit of Figure 6 associated with $\lambda_{0}$, we obtain a reduced circuit that provides a good approximation of the action of U(t) when the initial condition is the uniform state. Using Equations (3) and (30), the asymptotic overlap between $|\lambda_{0}\rangle$ and |ψ(0)〉 is

(39) $${\left|\langle \lambda_{0}|\psi \left(0\right)\rangle \right|}^{2}=\frac{1}{16N^{2}}+O\left(\frac{1}{N^{3}}\right),$$

which shows that asymptotically we can remove the term $\lambda_{0}|\lambda_{0}\rangle \langle \lambda_{0}|$ from Equation (28) because the initial condition is |ψ(0)〉. Consistently, ${\left|\langle \lambda^{+}|\psi \left(0\right)\rangle \right|}^{2}+{\left|\langle \lambda^{-}|\psi \left(0\right)\rangle \right|}^{2}\to 1$ in the asymptotic limit. The same behavior can be observed in many other graph classes, in which the asymptotic time-complexity of the search algorithm is determined by only two eigenvectors of the modified Hamiltonian.

**Figure 8:**
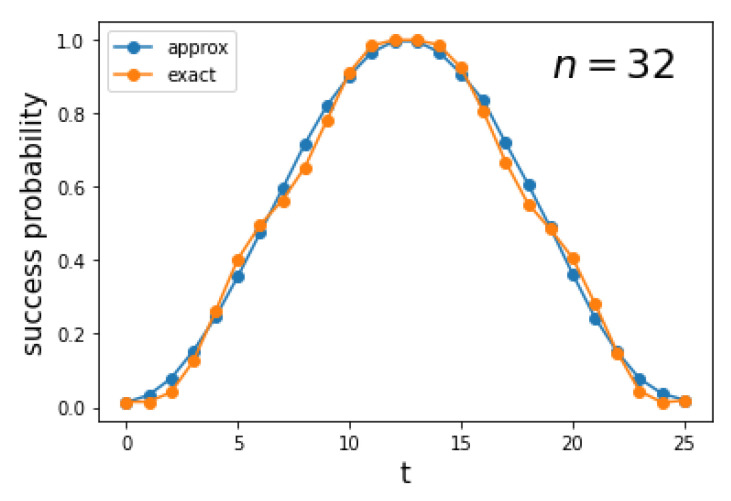
Success probability ${\left|\langle 0|\psi (t)\rangle \right|}^{2}$ as a function of the number of steps *t* for the search on $K_{n,n}$ with n=32. The orange curve is obtained using the exact decomposition (35) and the blue curve using the approximate circuit, both in Qiskit.

Figure 8 depicts the success probability ${\left|\langle 0|\psi (t)\rangle \right|}^{2}$ as a function of the number of steps *t* for a complete bipartite graph $K_{32,32}$. The orange curve is obtained using the implementation of the circuit of Figure 6 and the blue curve is obtained using the approximate circuit (without the gates associated with $\lambda_{0}$), both using Qiskit. The curves are remarkably close even with N=64. The optimal running time is given by Equation (A17), which is

(40) $$t_{\mathrm{opt}}=\left\lfloor \frac{\pi }{2}\sqrt{N}\right\rfloor$$

asymptotically. The blue curve is the square of a sinusoidal function, consistent with Equation (A16).

## 5. Hypercube

The *n*-dimensional hypercube $Q_{n}$ is a graph whose vertex set is labeled by the binary *n*-tuples and two vertices are adjacent if their Hamming distance is exactly one. The adjacency matrix is

(41) $$A=\sum_{j=1}^{n}X_{j},$$

where $X_{j}$ is the Pauli-*X* matrix acting on the *j*-th qubit. The computational basis of the Hilbert space associated with $Q_{n}$ is the set of all *n*-tuples and the dimension of the Hilbert space is $N=2^{n}$. The evolution operator of the CTQW on $Q_{n}$ with no marked vertex at time *t* is

(42) $$U\left(t\right)={\left(R_{x}\left(-2\gamma t\right)\right)}^{⊗n},$$

where $R_{x}\left(\theta \right)=\exp\left(-i\theta X/2\right)$.

In the next subsection, we address spatial search on the hypercube and obtain the optimal value of γ for the search algorithm.

### Spatial Search on Hypercube

To build the circuit of the spatial search algorithm on the hypercube, instead of taking the full Hamiltonian (1), we use an approximate Hamiltonian given by

(43) $$H_{\mathrm{approx}}=\lambda^{-}|\lambda^{-}\rangle \langle \lambda^{-}|+\lambda^{+}|\lambda^{+}\rangle \langle \lambda^{+}|,$$

where $\lambda^{\pm }$ are the eigenvalues of the first excited and ground states, respectively, of the exact Hamiltonian; $|\lambda^{\pm }\rangle$ are the associated normalized eigenvectors. We calculate a good approximation for $\lambda^{\pm }$ and $|\lambda^{\pm }\rangle$ by using the techniques of Appendix B. We disregard the remaining eigenvectors $|\lambda \rangle \notin \mathrm{span}\left\{|\lambda^{\pm }\rangle \right\}$ of the exact Hamiltonian because either they are associated with zero eigenvalues or $\left|\langle \lambda |\psi (0)\rangle \right|\to 0$ asymptotically, where |ψ(0)〉 is the initial condition (3).

**Figure 9:**
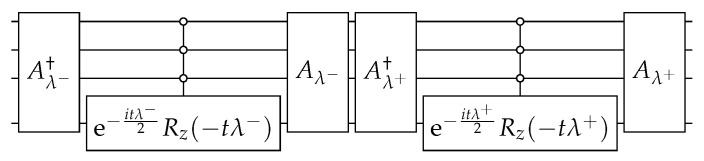
The circuit that implements U(t) (45) on the hypercube $Q_{n}$ with one marked vertex when n=4. The circuit of $A_{\lambda^{\pm }}$ is depicted in Figure 10.

The circuit that simulates the evolution operator

(44) $$U\left(t\right)=e^{-itH_{\mathrm{approx}}}$$

is depicted in Figure 9. To build this circuit, we replace (43) in (44) in order to obtain

(45) $$U\left(t\right)=A_{\lambda^{+}}R_{\lambda^{+}}A_{\lambda^{+}}^{†}A_{\lambda^{-}}R_{\lambda^{-}}A_{\lambda^{-}}^{†}$$

where

(46) $$R_{\lambda^{\pm }}=I+\left(e^{-it\lambda^{\pm }}-1\right)\left|0\rangle \langle 0\right|$$

and

(47) $$A_{\lambda^{\pm }}|0\rangle =|\lambda^{\pm }\rangle .$$

**Figure 10:**
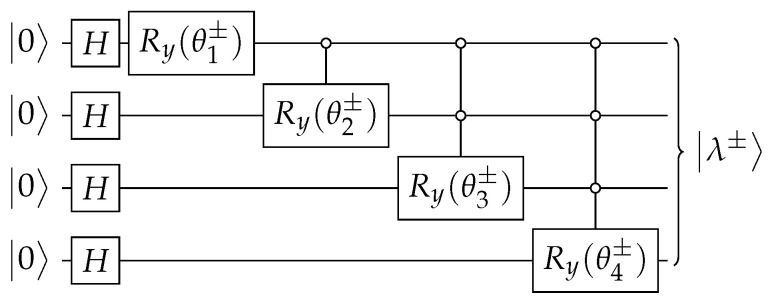
The circuit that implements $A_{\lambda^{\pm }}$ (47) when n=4. Angles $\theta_{k}^{\pm }$ are given by Equation (60).

The circuit of $A_{\lambda^{\pm }}$, which is depicted in Figure 10, is obtained using the techniques described in Appendix A and $|\lambda^{\pm }\rangle$ using Appendix B. In the next paragraphs, we give the details.

Since the characteristic polynomial of *A* (41) is

(48) ∏k=0nϕk+2k−nnk,

the eigenvalues are

(49) $$\phi_{k}=n-2k$$

for 0≤k≤n, with multiplicity nk. Since

(50) $$\tilde{A}|\ell \rangle =\left(n-2|\ell |\right)|\ell \rangle ,$$

where $\tilde{A}=H^{⊗n}AH^{⊗n}$ and |ℓ| is the Hamming weight of *ℓ*, the eigenvectors associated with eigenvalue (n−2k) are $H^{⊗n}|\ell \rangle$ such that |ℓ|=k. Define projector $P_{k}$ onto the $\phi_{k}$-eigenspace as

(51) $$P_{k}=\sum_{\begin{array}{c} \ell =0\\ |\ell |=k \end{array}}^{N-1}H^{⊗n}|\ell \rangle \langle \ell |H^{⊗n}.$$

Using these projectors, we obtain

(52) Pk|w〉2=1N2∑y=0N−1∑|ℓ|=k(−1)ℓ·y2=1Nnk.

Using Formulas (A12) and (A13), we obtain

(53) S1=12N∑k=1n1knk=1n+1n2+O1n3,

(54) S2=14N∑k=1n1k2nk=1n2+O1n3.

The asymptotic expansion of $S_{1}$ is described in Ref. [27] and of $S_{2}$ is obtained using the inequalities 14N∑k=1n1(k+1)(k+2)nk≤S2≤S12. Using (A14), we have $\gamma =S_{1}$, and therefore,

(55) $$\gamma =\frac{1}{n}+O\left(\frac{1}{n^{2}}\right).$$

Note that this optimal value of γ satisfies a weaker form of the locality condition discussed in Section 2.2, when compared to the optimal values of γ for $K_{N}$ and $K_{n,n}$. Using Equation (A15), we obtain

(56) $$\epsilon =\frac{1}{\sqrt{N}}+O\left(\frac{1}{n\sqrt{N}}\right).$$

and then using Equation (A11), we obtain

(57) $$\lambda^{\pm }=-1\pm \frac{1}{\sqrt{N}}+O\left(\frac{1}{N}\right).$$

Using Equation (A24), we obtain the following approximation for the components of the eigenvectors:

(58) $$\lambda^{\pm }\left(j\right)=\frac{1}{\sqrt{2}}\left(\delta_{jw}\mp \frac{1}{\sqrt{N}}\right).$$

Let us take w=0 (if w≠0, we must implement the modifications described at the end of Appendix A). Note that $\langle \lambda^{+}|\lambda^{-}\rangle =0$ and $\langle \lambda^{\pm }|\lambda^{\pm }\rangle =1\mp 2/\sqrt{N}$. The norms of these eigenvectors tend to 1 asymptotically. Since $|\lambda^{\pm }\rangle$ belongs to $\mathrm{span}\left\{|\alpha_{0}\rangle ,\ldots ,|\alpha_{n}\rangle \right\}$ and minding the normalizing factor in Equation (A3), Equation (A6) implies that $\alpha_{0}=\lambda^{\pm }\left(0\right)$ and $\alpha_{k}=\sqrt{2^{k-1}}\lambda^{\pm }\left(2^{k-1}\right)$ (1≤k≤n). Replacing $\alpha_{k}$ into Equation (A9), we obtain $\theta_{0}^{\prime \pm }=\pi /2$ and

(59) $$\theta_{k}^{\prime \pm }=\mp \arctan\frac{1}{\sqrt{1+2^{1-k}N}}.$$

for 1≤k≤n. Then, using Equation (A5), we obtain

(60) $$\theta_{k}^{\pm }=\mp 2\arctan\frac{1}{\sqrt{1+2^{k}}}-\frac{\pi }{2}$$

for 1≤k≤n. Using $\theta_{k}^{\pm }$, we obtain the circuit for $A_{\lambda^{\pm }}$ such that $A_{\lambda^{\pm }}|0\rangle =|\lambda^{\pm }\rangle$, which is depicted in Figure 10.

**Figure 11:**
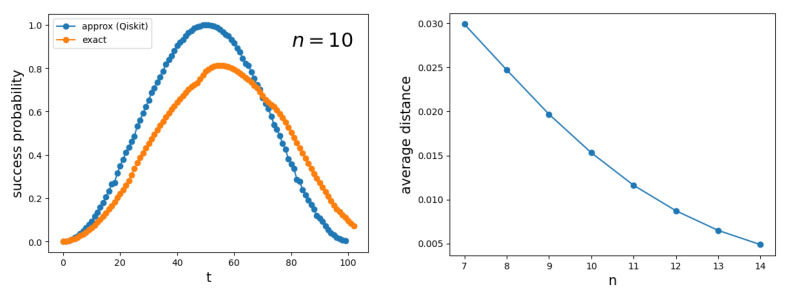
The first panel shows the success probability ${\left|\langle 0|\psi (t)\rangle \right|}^{2}$ as a function of the number of steps *t* for the search on $Q_{n}$ with n=10. The blue curve is obtained using the decomposition (45), which provides an approximation of the true success probability (orange curve), computed using the exact Hamiltonian (1). The second panel shows the average distance between the blue and orange curves as a function of the hypercube dimension.

The first panel of Figure 11 depicts the success probability ${\left|\langle 0|\psi (t)\rangle \right|}^{2}$ as a function of the number of steps *t* for a hypercube $Q_{10}$. The blue curve is obtained using the implementation of the circuit from Figure 9 in Qiskit, while the orange curve is generated using a numerical simulation of the exact Hamiltonian (1) with $\gamma =S_{1}$, implemented with the Hiperwalk package (https://hiperwalk.org/ (accessed on 19 March 2025)). The program used to produce these results is available at Hiperwalk’s GitHub (https://github.com/hiperwalk/hiperwalk/tree/master/examples/continuous_time) (accessed on 19 March 2025).

The optimal running time is given by Equation (A17), which is

(61) $$t_{\mathrm{opt}}=\left\lfloor \frac{\pi }{2}\sqrt{N}\right\rfloor$$

asymptotically. The blue curve exhibits a square-sinusoidal profile because it is derived using only the eigenvectors $|\lambda^{\pm }\rangle$, as shown in Equation (A16). As the dimension of the hypercube increases, the two curves converge, as illustrated in the second panel of Figure 11. This indicates that the blue curve provides a good approximation of both the running time and the success probability when *n* is sufficiently large.

## 6. Conclusions

In this work, we have described circuits that implement the evolution operator of the continuous-time quantum walk (CTQW)-based search algorithm on three graph classes: complete graphs, complete bipartite graphs, and hypercubes. We have implemented these circuits in Qiskit to compare them with the analytic evolution operators. For complete and complete bipartite graphs, the circuits implement the evolution operators exactly, modulo a global phase. For hypercubes, the circuit approximates the evolution operator, ensuring that the probability of finding the marked vertex closely matches that of the analytic operator when the hypercube dimension is sufficiently large.

The approximate implementation developed for hypercubes extends to a broader class of graphs, enhancing the versatility of CTQW-based search algorithms. This flexibility is particularly relevant in complex networks where exact implementations may be impractical. The framework described in [25] provides a method for determining the computational complexity of spatial search by CTQW on arbitrary graphs with multiple marked vertices, relying on two eigenvalues and two eigenvectors of the adjacency matrix. Our approximate implementation follows this framework, offering practical tools for implementing CTQW-based search algorithms on various graph structures.

As future work, we plan to pursue three directions: (1) apply our method to other graph classes, such as *N*-dimensional lattices; (2) explore the multi-marked vertex scenarios; and (3) explore potential applications of continuous-time quantum walks in quantum machine learning tasks, such as clustering or classification.

## Figures and Tables

**Figure A1:**
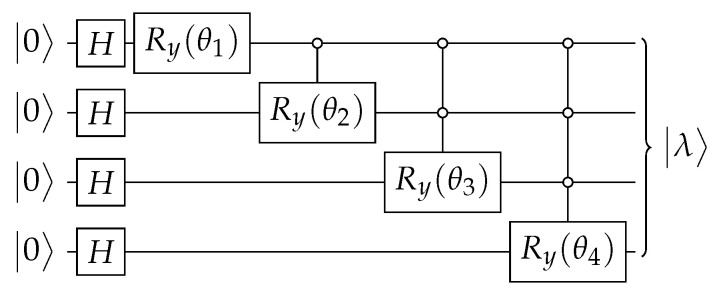
Ansatz for the circuit that implements an operator $A_{\lambda }$ with the property that $A_{\lambda }|0\rangle =|\lambda \rangle$ when n=4. Angles $\theta_{k}$ are obtained from Equations (A5) and (A9).

## Data Availability

Data are contained within the article.

## Abbreviations

The following abbreviations are used in this manuscript:

CTQW	Continuous-Time Quantum Walk

## Appendix A. State Preparation

There is a well-known recipe for obtaining a circuit of an operator *A* that outputs a generic state |λ〉 of *n* qubits when the input is |0〉—that is,

(A1) $$A_{\lambda }|0\rangle =|\lambda \rangle .$$

If |λ〉 has real entries and has many repeated entries, as discussed below, the Ansatz of Figure A1 provides a simpler circuit that has $O(n^{2})$ basic gates assuming reasonable restrictions on $\theta_{k}$. The goal is to obtain angles $\theta_{1},\ldots ,\theta_{n}$ that are used as parameters of the multi-controlled $R_{y}$ gates, where

(A2) $$R_{y}\left(\theta \right)=e^{-i\frac{\theta }{2}Y}=\left(\begin{array}{cc} \cos\frac{\theta }{2} & -\sin\frac{\theta }{2}\;\\ \sin\frac{\theta }{2} & \cos\frac{\theta }{2} \end{array}\right).$$

Define $|\alpha_{0}\rangle =|0\rangle$ and

(A3) $$|\alpha_{k}\rangle =\frac{1}{\sqrt{2^{k-1}}}\sum_{j=2^{k-1}}^{2^{k}-1}|j\rangle ,$$

for 1≤k≤n, where $\left\{|j\rangle :0\leq j\leq 2^{n}-1\right\}$ is the computational basis. If $\left|\lambda \rangle \in \mathrm{span}\{|\alpha_{0}\rangle ,\ldots ,|\alpha_{n}\rangle \right\}$, then we can find $\theta_{1},\ldots ,\theta_{n}$ such that |λ〉 is the output of the circuit of Figure A1. To show this statement, we use the fact that the output of the circuit is

(A4) $$|\lambda \rangle =\sum_{k=0}^{n}\sin\theta_{k}^{\prime }\prod_{j=k+1}^{n}\cos\theta_{j}^{\prime }|\alpha_{k}\rangle ,$$

where

(A5) $$\theta_{k}=2\theta_{n-k+1}^{\prime }-\frac{\pi }{2}$$

for 1≤k≤n and $\theta_{0}^{\prime }=\pi /2$. Assuming that

(A6) $$|\lambda \rangle =\sum_{k=0}^{n}\alpha_{k}|\alpha_{k}\rangle ,$$

we obtain the system of equations

(A7) $$\alpha_{k}=\sin\theta_{k}^{\prime }\prod_{j=k+1}^{n}\cos\theta_{j}^{\prime },$$

for 0≤k≤n in terms of variables $\theta_{1}^{\prime },\ldots ,\theta_{n}^{\prime }$. The solution is obtained in a recursive way starting with equation $\alpha_{n}=\sin\theta_{n}^{\prime }$ and decreasing the index *k* of Equation (A7). We obtain

(A8) $$\sin\theta_{k}^{\prime }=\frac{\alpha_{k}}{\sqrt{1-\sum_{j=k+1}^{n}{\alpha_{j}}^{2}}},$$

and then

(A9) $$\theta_{k}^{\prime }=\arctan\frac{\alpha_{k}}{\sqrt{1-\sum_{j=k}^{n}{\alpha_{j}}^{2}}}.$$

To conclude, the circuit of Figure A1 outputs (A6) when angles $\theta_{k}$ are given by Equations (A5) and (A9).

Note that the entry $\alpha_{0}$ of vector |λ〉 in (A6) plays a special role in the spatial search algorithm, because it is associated with the label of the marked vertex. If the label of the marked vertex is *ℓ* (assume that *ℓ* is even by now), we want to define $|\alpha_{\ell }\rangle =|\ell \rangle$. In this case, we have to change some empty controls of the $R_{y}\left(\theta \right)$-gates in the circuit of Figure A1 into solid controls. The recipe is as follows. Let us focus on the last gate of Figure A1, which has n−1 empty controls. We want this gate to be activated if the input is *ℓ*. We change from empty to solid all controls associated with bits 1 of *ℓ*. The same modifications must be applied to the preceding gates. On the other hand, if *ℓ* is odd, we have to do an extra change, not in terms of converting further empty into solid controls, but by changing $\theta_{n}^{\prime }$ into $\pi /2-\theta_{n}^{\prime }$.

## Appendix B. Eigenvectors of the Modified Hamiltonian

Suppose that the distinct eigenvalues of the adjacency matrix *A* are $\phi_{0}>\phi_{1}>\cdots >\phi_{q}$. Define $P_{\ell }$ as the orthogonal projector onto the eigenspace of *A* associated with eigenvalue $\phi_{\ell }$ for 0⩽ℓ⩽q, so that

(A10) $$A=\sum_{\ell =0}^{q}\phi_{\ell }P_{\ell }.$$

Let us assume that the asymptotic dynamics of the search algorithm depends only on two eigenvectors $|\lambda^{\pm }\rangle$ of *H* (1) associated with eigenvalues $\lambda^{\pm }$ closest to $-\gamma \phi_{0}$, such as

(A11) $$\lambda^{\pm }=-\gamma \phi_{0}\pm \epsilon +O\left(\epsilon^{2}\right),$$

where 2ϵ>0 is the spectral gap. Refs. [25,28] shows that the computational complexity of the search algorithm with one marked vertex *w* is determined by two sums given by

(A12) $$S_{1}=\sum_{\ell =1}^{q}\frac{{∥P_{\ell }|w\rangle ∥}^{2}}{\phi_{0}-\phi_{\ell }}$$

and

(A13) $$S_{2}=\sum_{\ell =1}^{q}\frac{{∥P_{\ell }|w\rangle ∥}^{2}}{{\left(\phi_{0}-\phi_{\ell }\right)}^{2}},$$

and the asymptotic value of γ is obtained as

(A14) $$\gamma =S_{1}$$

and

(A15) $$\epsilon =\frac{S_{1}∥P_{0}|w\rangle ∥}{\sqrt{S_{2}}}.$$

Asymptotically, the success probability ${\left|\langle 0|\psi (t)\rangle \right|}^{2}$ is

(A16) $$p\left(t\right)=4{\left|\langle \lambda^{+}|\psi \left(0\right)\rangle \right|}^{2}{\left|\langle w|\lambda^{+}\rangle \right|}^{2}\sin^{2}\epsilon t,$$

and the optimal running time is

(A17) $$t_{\mathrm{opt}}=\frac{\pi }{2\epsilon }.$$

To calculate the success probability, we need two extra quantities that are (1) the overlap between $|\lambda^{\pm }\rangle$ and the marked vertex, which is

(A18) $$\langle w|\lambda^{\pm }\rangle =\frac{S_{1}}{\sqrt{2S_{2}}},$$

and (2) the overlap between $|\lambda^{\pm }\rangle$ and the initial state, which is

(A19) $$\langle \psi \left(0\right)|\lambda^{\pm }\rangle =\mp \frac{1}{\sqrt{2N}∥P_{0}|w\rangle ∥}.$$

Now, we are ready to calculate an asymptotic expression for $\langle j|\lambda^{\pm }\rangle$, that is, the (j+1)-th entry of $|\lambda^{\pm }\rangle$. These calculations go beyond the results of Refs. [25,28]. Let us assume that the initial condition is a linear combination of $|\lambda^{+}\rangle$ and $|\lambda^{-}\rangle$ in the asymptotic limit. Using the completeness relation, we obtain

(A20) $$\langle j|\psi (0)\rangle =\langle \lambda^{+}|\psi \left(0\right)\rangle \;\lambda^{+}\left(j\right)+\langle \lambda^{-}|\psi \left(0\right)\rangle \;\lambda^{-}\left(j\right),$$

where $\lambda^{+}\left(j\right)=\langle j|\lambda^{+}\rangle$ and $\lambda^{-}\left(j\right)=\langle j|\lambda^{-}\rangle$ are the unknowns. We obtain a second independent equation in these unknowns by sandwiching *H* as follows:

(A21) $$\langle j|H|\psi (0)\rangle =\lambda^{+}\langle \lambda^{+}|\psi \left(0\right)\rangle \;\lambda^{+}\left(j\right)+\lambda^{-}\langle \lambda^{-}|\psi \left(0\right)\rangle \;\lambda^{-}\left(j\right).$$

Using Equations (1) and (A10), we obtain

(A22) $$\langle j|H|\psi (0)\rangle =-S-\frac{\delta_{jw}}{\sqrt{N}},$$

where

(A23) $$S=\gamma \sum_{\ell }\phi_{\ell }\langle j|P_{\ell }|\psi \left(0\right)\rangle ,$$

and assuming that |ψ(0)〉 is the uniform state. The solution of the system of Equations (A20) and (A21) is

(A24) $$\begin{array}{cc} \lambda^{\pm }\left(j\right) & =\frac{\mp 1}{2\epsilon \sqrt{N}\langle \lambda^{\pm }|\psi \left(0\right)\rangle }\left(\sqrt{N}S+\delta_{jw}+\lambda^{\mp }\right). \end{array}$$

The circuit of $A_{\lambda^{\pm }}$ such that $A_{\lambda^{\pm }}|0\rangle =|\lambda^{\pm }\rangle$ can be obtained using the techniques of Appendix A.
